# Phenolics: From Chemistry to Biology

**DOI:** 10.3390/molecules14062202

**Published:** 2009-06-17

**Authors:** David M. Pereira, Patrícia Valentão, José A. Pereira, Paula B. Andrade

**Affiliations:** 1 REQUI*M*TE/Department of Pharmacognosy, Faculty of Pharmacy, Porto University, R. Aníbal Cunha, 164, 4050-047 Porto, Portugal; 2 CIMO/Escola Superior Agrária, Instituto Politécnico de Bragança, Campus de Sta. Apolónia, Apartado 1172, 5301-855 Bragança, Portugal

**Keywords:** phenolics, chemistry, bioactivities

## Abstract

In recent years, few classes of natural products have received as much attention as phenolics and polyphenols. This special issue of *Molecules*, “Phenolics and Polyphenolics”, is a remarkable confirmation of this trend. Several aspects related to phenolics chemistry, comprising the several classes, will be discussed. In addition, the increasing interest in phenolics’ biological activities is covered, and several works addressing this matter are referred.

## A world of phenolics

**Figure 1:**
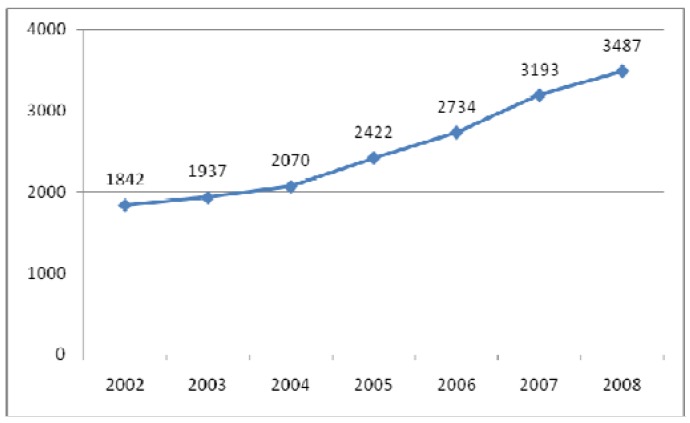
Evolution in the number of papers with the keyword “phenolics” (Scopus, June 2009).

This special issue of Molecules, “Phenolics and Polyphenolics”, will focus on the vast world of polyphenols, from their rich chemistry to their extensive list of biological properties. Without a doubt, this area of knowledge has experienced an increasing popularity in the past years, as represented in Figure 1.

Polyphenols are among the most widespread class of metabolites in nature, and their distribution is almost ubiquitous. It is estimated that 100,000 to 200,000 secondary metabolites exist [1] and some 20% of the carbon fixed by photosynthesis is channeled into the phenylpropanoid pathway, thus generating the majority of the natural-occurring phenolics, such as flavonoids and stilbenes [1,2]. Although strictly speaking monophenols, such as *p*-coumaric acid, are not polyphenols, they share however with these many of their properties and characteristics, being thus known as “functional polyphenols” [3].

**Figure 2:**
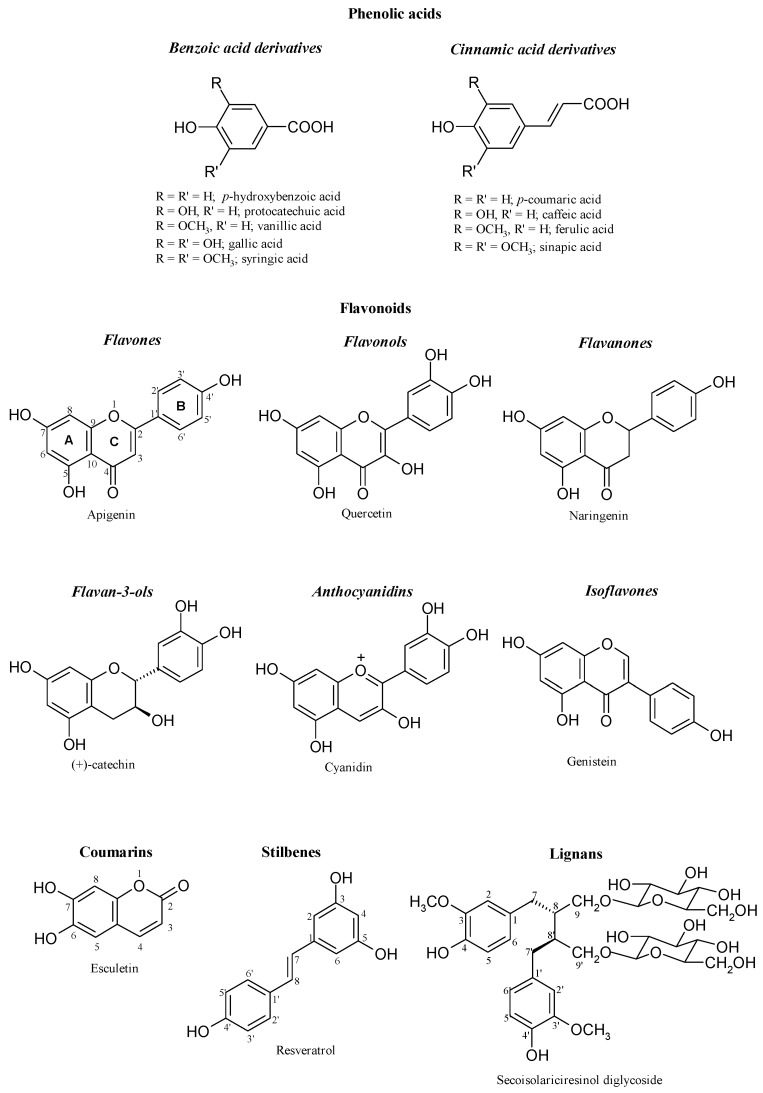
Main classes of phenolics (from [4]).

Although a large variety of plant phenols exists, most of these compounds arise from a common origin: the amino acids phenylalanine or tyrosine. These aminoacids are deaminated to cinnamic acids, which enter the phenylpropanoid pathway. A key step in this biosynthetic route is the introduction of one or more hydroxyl groups into the phenyl ring. As result, these compounds are derived from a common carbon skeleton building block: the C_6_-C_3_ phenylpropanoid unit. Biosynthesis, according to this pathway, produces the large variety of plant phenols: cinnamic acids (C_6_-C_3_), benzoic acids (C_6_-C_1_), flavonoids (C_6_-C_3_-C_6_), proanthocyanidins [(C_6_-C_3_-C_6_)*_n_*], coumarins (C_6_-C_3_), stilbenes (C_6_-C_2_-C_6_), lignans (C_6_-C_3_-C_3_-C_6_) and lignins [(C_6_-C_3_)*_n_*] [4]. The main phenolic structures are shown in Figure 2.

## General antioxidant mechanisms of phenolics

Phenolics are able to act as antioxidants in a number of ways. Phenolic hydroxyl groups are good hydrogen donors: hydrogen-donating antioxidants can react with reactive oxygen and reactive nitrogen species [5,6,7,8,9,10,11] in a termination reaction, which breaks the cycle of generation of new radicals. Following interaction with the initial reactive species, a radical form of the antioxidant is produced, having a much greater chemical stability than the initial radical. The interaction of the hydroxyl groups of phenolics with the π-electrons of the benzene ring gives the molecules special properties, most notably the ability to generate free radicals where the radical is stabilized by delocalization. The formation of these relatively long-lived radicals is able to modify radical-mediated oxidation processes [12].

The antioxidant capacity of phenolic compounds is also attributed to their ability to chelate metal ions involved in the production of free radicals [13]. However, phenolics can act as pro-oxidants by chelating metals in a manner that maintains or increases their catalytic activity or by reducing metals, thus increasing their ability to form free radicals [14].

Phenolic structures often have the potential to strongly interact with proteins, due to their hydrophobic benzenoid rings and hydrogen-bonding potential of the phenolic hydroxyl groups. This gives phenolics the ability to act as antioxidants also by virtue of their capacity to inhibit some enzymes involved in radical generation, such as various cytochrome P_450_ isoforms, lipoxygenases, cyclooxygenase and xanthine oxidase [12,15].

Additionally, synergistic effects of phenolics with other antioxidants, namely ascorbic acid, β-carotene and α-tocopherol [14], and regulation of intracellular glutathione levels have also been described [4,16].

## Flavonoids

Flavonoids are characterized by a phenylbenzopyran chemical structure. The general structure includes a C_15_ (C_6_-C_3_-C_6_) skeleton joined to a chroman ring (benzopyran moiety). The heterocyclic benzopyran ring is known as the C ring, the fused aromatic ring as the A ring, and the phenyl constituent as the B ring. The A ring can be of two types: a phloroglucinol type that is *meta*-trihydroxylated, or a resorcinol type that is *meta*-dihydroxylated [17,18]. The B ring can be monohydroxylated, *ortho*-dihydroxylated or vicinal-trihydroxylated. The center heterocycle most commonly exists in one of three forms: pyran, pyrilium, or γ-pyrone [19].

According to the position of the aromatic ring to the benzopyrane moiety, flavonoids can be grouped in four classes: major flavonoids (2-phenylbenzopyrans), isoflavonoids (3-benzopyrans), neoflavonoids (4-benzopyranes) and minor flavonoids.

In plants, these compounds occur in nearly all species, usually as a result of their UV screening properties, thus constituting a protection for the plant [20]. Also, their ability to attract pollinators is well established [21].

Flavonoid identification and quantification techniques, such as HPLC-DAD or LC-MS, are diverse and some of them explore their UV absorption properties, which usually allow distinguishing among different classes. For compounds for which authentic standards exist, HPLC-DAD, by providing both retention time and UV spectrum is enough for positive identification. However, when no commercial standard is available, a very common situation, techniques that yield more structural information are required. In this field, LC-MS constitutes a valuable tool for flavonoids’ identification [22].

Increasingly, flavonoids are becoming the subject of medical research. They have been reported to possess many useful properties, including anti-inflammatory, oestrogenic, enzyme inhibition, antimicrobial, antiallergic, vascular and cytotoxic antitumour activity [23], but the antioxidant activity is, without a doubt, the most studied one attributed to flavonoids. This well established antioxidant activity of flavonoids is also responsible for other biological activities in which the prevention of oxidative stress is beneficial. For example, the anticancer activity of some compounds is due to their ability to scavenge free radicals, thus avoiding the early stages of cancer promotion. Besides this mechanism, flavonoids have also been reported to act as anticancer agents *via* regulation of signal transduction pathways of cell growth and proliferation, suppression of oncogenes and tumor formation, induction of apoptosis, modulation of enzyme activity related to detoxification, oxidation and reduction, stimulation of the immune system and DNA repair, and regulation of hormone metabolism [24].

In the past few years we have witnessed the establishment of other flavonoid classes as potent molecules for the treatment of other pathologies that do not involve these compounds’ antioxidant properties. This is the case of some isoflavones, whose estrogen-like capacity is now well established. The activity of these compounds is related with their similarity to estradiol estrogen. Genistein and daidzein have demonstrated to be promising molecules for the treatment of conditions in which the agonist effect in estrogen receptors is beneficial, such as menopause conditions. In fact, several preparations containing these compounds, mainly soya-derived, are now used in therapeutics [25].

The venoprotective properties of flavonoids are also explored in several formulations for the enhancement of micro-circulation in pathological conditions in which this function is compromised.

## Cinnamic acids

L-Phenylalanine and L-tyrosine, as C_6_C_3_ building blocks, are precursors for a wide range of natural products. In plants, a frequent first step is the elimination of ammonia from the side-chain to generate the appropriate *trans-*(*E*)-cinnamic acid. In the case of phenylalanine, this would give cinnamic acid, whilst tyrosine could yield 4-coumaric acid (*p*-coumaric acid). All plants appear to have the ability to deaminate phenylalanine *via* phenylalanine ammonia lyase (PAL) enzyme, but the corresponding transformation of tyrosine is more restricted, being mainly limited to members of the grass family (the Graminae/Poaceae). The most representative cinnamic acid is caffeic acid, which occurs in fruits, vegetables and coffee, mainly as an ester with quinic acid (chlorogenic acid or 5-caffeoylquinic acid) [13,4].

The antioxidant activity of phenolic acids is related to the number and position of hydroxyl groups in the molecule. The antioxidant efficiency of mono-phenols is strongly enhanced by the introduction of a second hydroxyl group at the *ortho-* or *para-* positions, and is increased by one or two methoxy substitutions in *ortho-* position with respect to the hydroxyl group [26,27].

While flavonoids present several physical properties, which have made photodiode array detection a very useful approach, phenolic acids, although they may be identified by HPLC-DAD and LC-MS, are best analysed by GC-MS, given their volatility. Contrarily to flavonoids, a great number of phenolic acids are commercially available, thus allowing a definitive identification. But this methodology is not applied to phenolic acids derivatives with quinic or tartaric acids, or glycosylated ones. For these compounds the best analytic technique is LC-MS.

As it happens with most polyphenols, cinnamic acids also exhibit strong antioxidant properties. This activity can be expressed in several ways. For instance, 1,5-dicaffeoylquinic acid has been revealed to be an hepatoprotector when challenged by carbon tetrachloride, a mechanism that involves, among others, radical scavenging [20].

## Lignin and lignans

Cinnamic acids also feature in the pathways to other metabolites based on C_6_C_3_ building blocks. An important example is the plant polymer lignin, a strengthening material for the plant cell wall which acts as a matrix for cellulose microfibrils. Lignin represents a vast reservoir of aromatic materials, mainly untapped because of the difficulties associated with release of these metabolites. The action of wood-rotting fungi offers the most effective way of making these useful products more accessible. Lignin is formed by phenolic oxidative coupling of hydroxycinnamoyl alcohol monomers, brought about by peroxidase enzymes. The most important of these monomers are 4-hydroxycinnamoyl alcohol (*p*-coumaroyl alcohol), coniferyl alcohol, and sinapoyl alcohol, though the monomers used vary according to the plant type [27]. In what concerns to bioactivity, it is poorly studied and aside from antioxidant activity, the number of studies on this matter are rather scarce. Nevertheless, tyrosine inhibiting activity has been described [28].

Lignans are organic compounds resultant from the establishment of a link between β carbons of the side chain of two 1-phenylpropane derivatives (8-8’ link). Numerous compounds possess cytostatic and antimitotic properties, perhaps the most widely known bioactivity. However, only hemisynthetic derivatives of podophylotoxin (obtained from the rhizome of *Podophyllum peltatum*) have been explored in therapeutics. In addition, several other properties have been reported for lignans: inhibition of AMPc phosphodiesterase and of enzymes from the respiratory chain and antihypertensive activity [20].

## Anthocyanins

Anthocyanins are water soluble plant pigments, usually with molecular weights ranging from 400 to 1,200, and responsible for the blue, purple and red colors of many plant tissues [29]. These compounds are glycosylated polyhydroxy- and polymethoxy-derivatives of 2-phenylbenzopyrylium (flavylium) salts. The most common sugars are glucose, galactose, rhamnose and arabinose. These sugars are usually linked at the 3 position of the C ring or at the 5 and 7 positions of the B ring, occurring as mono-, di- or tri-saccharide forms. Although very rare, glycosylation at the 3’, 4’, or 5’ positions of the B ring is also possible [30]. Despite the knowledge of about 17 anthocyanidins (anthocyanin aglycones), only six of them are ubiquitously distributed in nature: cyanidin, delphinidin, petunidin, peonidin, pelargonidin and malvidin.

With the exceptions of 3-deoxyanthocyanidins and their derivatives, there is always a glycosyl group in C-3, which means that aglycones are rarely found in nature. The sugar moiety may be acylated by aromatic acids, mostly hydroxycinnamic acids (caffeic, ferulic, *p*-coumaric or sinapic acids) and sometimes by aliphatic acids, namely malonic and acetic acids. These acyl moieties are usually linked to the sugar at C-3 [31].

The multiple possibilities regarding the identity and position of sugars and acyl moieties, as well as the position and number of hydroxy and methoxy groups on the anthocyanidin skeleton, gives rise to a great number of compounds, with over 600 anthocyanins being known today [32].

In the past few years, more attention has been given to the study of adducts between anthocyanins and several other compounds, such as organic acids, either natural-occurring or synthetic. An example is that described by Mateus *et al*., who identified a new pigment, the structure of which corresponded to a pyruvic acid adduct of malvidin 3-glucoside linked to a vinyl phenol group [33].

An unusual *C*-glycosyl-anthocyanin has been described by Tatsuzawa and colleagues from the flowers of the toad lily, *Tricyrtis formosana* (Liliaceae) [34]. This species remains the only recorded source of C-glycosylanthocyanins. Recently, significant anti-cancer properties of some anthocyanins against a range of cell lines have been described [35].

## Tannins

The designation of tannin includes compounds of two distinct chemical groups: hydrolysable tannins (polymers of ellagic acid, or of gallic and ellagic acids, with glucose) [20] and condensed tannins, which result from the condensation of monomers of flavan-3-ol units [36].

Tannins are substances that are able to combine with proteins of animal hide preventing their putrefaction and converting them into leather. This ability comprises all kinds of proteins and, therefore, enzymes are included.

Given their relationship to phenolic acids and flavonoids, their antioxidant properties are not a surprise: they exert their antioxidant activity by scavenging free radicals, chelating trace metals and by binding proteins with suppression of their enzymatic activity [4]. Yokozawa *et al.* [37] showed that the scavenging activity of tannins increases with an increase in the number of galloyl groups and molecular weight and in the presence of an *ortho*-dihydroxy structure: the hydroxyl groups are responsible for the chelating and radical scavenging properties of these compounds.

While sharing some antioxidant activities with other phenolics, recent works [38,39] have described the capacity of tannins to enhance glucose uptake and inhibit adipogenesis, thus being potential drugs for the treatment of non-insulin dependent diabetes mellitus.

As it was said to flavonoids, the antioxidant properties of tannins are equally responsible for other interesting biological properties. Flavan-3-ols are thought to interfere in the pathogenesis of cardiovascular disease *via* several mechanisms: antioxidative, antithrombogenic, and anti-inflammatory. In particular, proanthocyanidins and flavan-3-ol monomers aid in lowering plasma cholesterol levels, inhibit LDL oxidation, and activate endothelial nitric oxide synthase to prevent platelet adhesion and aggregation that contribute to blood clot formation [24,40].

## Coumarins

To this days, around 1300 coumarins are known, with all of them being derivatives of 5,6-benzo-2-pirone (*α*-chromone) (with OH, OCH_3_ or CH_3_ substituents on the benzoic ring.). In addition to simple coumarins, *C*-prenylated and *O*-prenylated forms exist.

As derivatives of simple coumarins, other compounds are known, such as furanocoumarins, which include a furanic ring, linear pyranocoumarins, angular pyranocoumarins, dimeric coumarins, of which dicoumarol is an example and also furanochromones [42].

Historically, the ability of dicoumarol to inhibit blood clotting, that later led to the development of the anticoagulant drug warfarin, was the first call to this class of compounds’ biological properties. Several biological activities have been reported in natural-occurring coumarins, from photo sensitizers to vasodilatation. Recently, the interest has been given to synthetic derivatives of coumarins, such as fluorinated and 1-azo coumarins, which displayed moderate analgesia properties, and excellent anti-inflammatory and anti-microbial activities [43].

As demonstrated in this special issue of Molecules, phenolic compounds, themselves, or present in natural matrices, are object of profound interest, in what concerns their chemistry and their interesting biological and pharmacological properties.

## References

[1] Metcalf R.L. (1987). Plant volatiles as insect attractants. CRC Crit. Rev. Plant Sci..

[2] Ralston L., Subramanian S., Matsuno M., Yu O. (2005). Partial reconstruction of flavonoid and isoflavonoid biosynthesis in yeast using soybean type I and type II chalcone isomerases. Plant Physiol..

[3] Dixon R.A. (2004). Phytoestrogens. Annu. Rev. Plant Physiol. Plant Mol. Biol..

[4] Seabra R.M., Andrade P.B., Valentão P., Fernandes E., Carvalho F., Bastos M.L., Fingerman M., Nagabhushanam R. (2006). Biomaterials from Aquatic and Terrestrial organisms.

[5] Valentão P., Fernandes E., Carvalho F., Andrade P.B., Seabra R.M., Bastos M.L. (2003). Hydroxyl radical and hypochlorous acid scavenging activity of small centaury (*Centaurium erythraea*) infusion. A comparative study with green tea (*Camellia sinensis*). Phytomedicine.

[6] Valentão P., Fernandes E., Carvalho F., Andrade P.B., Seabra R.M., Bastos M.L. (2002). Antioxidative properties of cardoon (*Cynara cardunculus* L.) infusion against superoxide radical, hydroxyl radical and hypochlorous acid. J. Agric. Food Chem..

[7] Valentão P., Fernandes E., Carvalho F., Andrade P.B., Seabra R.M., Bastos M.L. (2002). Antioxidant activity of *Hypericum androsaemum* infusion: scavenging activity against superoxide radical, hydroxyl radical and hypochlorous acid. Biol. Pharm. Bull..

[8] Valentão P., Fernandes E., Carvalho F., Andrade P.B., Seabra R.M., Bastos M.L. (2002). Studies on the antioxidant activity of *Lippia citriodora* infusion: scavenging effect on superoxide radical, hydroxyl radical and hypochlorous acid. Biol. Pharm. Bull..

[9] Heim K.E., Tagliaferro A.R., Bobilya D.J. (2002). Flavonoid antioxidants: chemistry, metabolism and structure-activity relationships. J. Nutrit. Biochem..

[10] Payá M., Halliwell B., Hoult J.R.S. (1992). Interactions of a series of coumarins with reactive oxygen species. Scavenging of superoxide, hypochlorous acid and hydroxyl radicals. Biochem. Pharmacol..

[11] Choi H.R., Choi J.S., Han Y.N., Bae S.J., Chung H.Y. (2002). Peroxynitrite scavenging activity of herb extracts. Phytother. Res..

[12] Parr A.J., Bolwell J.P. (2002). Phenols in the plant and in man. The potential for possible nutritional enhancement of the diet by modifying the phenols content or profile. J. Sci. Food Agric..

[13] Yang C.S., Landau J.M., Huang M.-T., Newmark H.L. (2001). Inhibition of carcinogenesis by dietary polyphenolic compounds. Annu. Rev. Nutr..

[14] Croft K.D. (1998). The chemistry and biological effects of flavonoids and phenolic acids. Ann. N. Y. Acad. Sci..

[15] Cos P., Ying L., Calomme M., Hu J.P., Cimanga K., Poel B.V., Pieters L., Vlietinck A.J., Berghe D.V. (1988). Structure-activity relationship and classification of flavonoids as inhibitors of xanthine oxidase and superoxide scavengers. J. Nat. Prod..

[16] Myhrstad M.C.W., Carlsen H., Nordström O., Blomhoff R., Moskaug J.Ø. (2002). Flavonoids increase the intracellular glutathione level by transactivation of the γ-glutamylcysteine synthetase catalytical subunit promoter. Free Radic. Biol. Med..

[17] Ribereau-Gayon P. (1972). Plant phenolics.

[18] Haslam E. (1998). Practical Polyphenols: From structure to molecular recognition and physiological action.

[19] Aron P.M., Kennedy J.A. (2008). Flavan-3-ols: Nature, occurrence and biological activity. Mol. Nutr. Food Res..

[20] Bruneton J. (1999). Pharmacognosie: phytochimie, plantes médicinales.

[21] Ferreres F., Pereira D.M., Valentão P., Andrade P.B., Seabra R.M., Sottomayor M. (2008). New phenolic compounds and antioxidant potential of *Catharanthus roseus*. J. Agric. Food Chem..

[22] Andrade P.B., Pereira D.M., Ferreres F., Valentão P. (2008). Recent trends in high throughput analysis and antioxidant potential screening for phenolics. Curr. Pharm. Anal..

[23] Cushnie T.P.T, Lamb A.J. (2005). Antimicrobial activity of flavonoids. Int. J. Antimicrob. Agents.

[24] Aron P.M., Kennedy J.A. (2008). Flavan-3-ols: Nature, occurrence and biological activity. Mol. Nutr. Food Res..

[25] Dixon R.A., Ferreira D. (2002). Genistein. Phytochemistry.

[26] Fukumoto L.R., Mazza G. (2000). Assessing antioxidant and prooxidant activities of phenolic compounds. J. Agric. Food Chem..

[27] Dewick P.M. (2002). Medicinal natural products: a biosynthetic approach.

[28] Azhar-ul-Haq, Malik A., Khan M.T.H., Anwar-Ul-Haq, Khan S.B., Ahmad A., Choudhary M.I. (2006). Tyrosinase inhibitory lignans from the methanol extract of the roots of *Vitex negundo* Linn. and their structure-activity relationship. Phytomedicine.

[29] Prior R.L., Wu X. (2006). Anthocyanins: Structural characteristics that result in unique metabolic patterns and biological activities. Free Radic. Res..

[30] Wu X., Prior R.L. (2005). Identification and characterization of anthocyanins by HPLC-ESI-MS/MS in common foods in the United States: Vegetables, nuts and grains. J. Agric. Food Chem..

[31] Harborne J.B. (1964). Plant polyphenols – XI. The structure of acylated anthocyanins. Phytochemistry.

[32] Anderson O.M. (2002). Anthocyanin occurrences and analysis. Proceedings of the International Workshop on Anthocyanins: Research and Development of Anthocyanins.

[33] Mateus N., Oliveira J., Pissarra J., González-Paramás A.M., Rivas-Gonzalo J.C., Santos-Buelga C., Silva A.M.S., de Freitas V. (2006). A new vinylpyranoanthocyanin pigment occurring in aged red wine. Food Chem..

[34] Tatsuzawa F., Saito N., Miyoshi K., Shinoda K., Shigihara A., Honda T. (2004). Diacylated 8-*C*-glucosylcyanidin 3-glucoside from the flowers of *Tricyrtis formosana*. Chem. Pharm. Bull..

[35] Zhang Y., Seeram N.P., Lee R., Feng L., Heber D. (2008). Isolation and identification of strawberry phenolics with antioxidant and human cancer cell antiproliferative properties. J. Agric. Food Chem..

[36] Waterhouse A. (2002). Wine phenolics. Ann. N Y Acad. Sci..

[37] Yokozawa T., Chen C.P., Dong E., Tanaka T., Nonaka G.-I., Nishioka I. (1998). Study on the inhibitory effect of tannins and flavonoids against the 1,1-diphenyl-2-picrylhydrazyl radical. Biochem. Pharmacol..

[38] Muthusamy V.S., Anand S., Sangeetha K.N., Sujatha S., Lakshmi B.A.B.S. (2008). Tannins present in *Cichorium intybus* enhance glucose uptake and inhibit adipogenesis in 3T3-L1 adipocytes through PTP1B inhibition. Chem. Biol. Interact..

[39] Liu F., Kim J., Li Y., Liu X., Li J., Chen X. (2001). An extract of *Lagerstroemia speciosa* L. has insulin-like glucose uptake-stimulatory and adipocyte differentiation-inhibitory activities in 3T3-L1 cells. J. Nutr..

[40] Bagchi D.B., Sen C.K., Ray S.D., Das D.K., Bagchi M., Preuss H.G., Vinson J.A. (2003). Molecular mechanisms of cardioprotection by a novel grape seed proanthocyanidin extract. Mutat. Res..

[41] Havsteen B.H. (2002). The biochemsitry and medical significance of the flavonoids. Pharmacol. Therap..

[42] Proença da Cunha A. (2005). Farmacognosia e Fitoquímica.

[43] Kalkhambkar R.G., Kulkarni G.M., Kamanavalli C.M., Premkumar N., Asdaq S.M.B., Sun C.M. (2008). Synthesis and biological activities of some new fluorinated coumarins and 1-aza coumarins. Eur. J. Med. Chem..

